# Ivabradine Prevents Heart Rate Acceleration in Patients with Chronic Obstructive Pulmonary Disease and Coronary Heart Disease after Salbutamol Inhalation

**DOI:** 10.3390/ph5040398

**Published:** 2012-04-16

**Authors:** Rustem Zulkarneev, Naufal Zagidullin, Guzel Abdrahmanova, Uta C. Hoppe, Shamil Zagidullin

**Affiliations:** 1 Bashkir State Medical University, Lenin Str. 3, Ufa 450000, Russia; 2 Department of Internal Medicine II, Paracelsus Medical University, Salzburg 5020, Austria

**Keywords:** ivabradine, COPD, CHD, salbutamol

## Abstract

Accelerated sinus rhythm is an important side effect of inhaled salbutamol which is especially harmful in patients with chronic obstructive pulmonary disease (COPD) and coronary heart disease (CHD). Cross-over, randomized, open label study design. 20 patients (18 males and two females) with COPD stage II–IV and comorbide CHD NYHA class I–III were included. Spirometry with 400 mg salbutamol inhalation was performed at two consecutive days of the study. Patients in group I were prescribed 5 mg ivabradine per os 3 h before salbutamol inhalation solely on the first day of the study and patients of group II received 5 mg ivabradine only on the second day of the study. Salbutamol caused a significant increase of HR by 5.5 bpm (95% CI 0.8; 10.2, *p* < 0.03). After ivabradine ingestion salbutamol did not change HR significantly by −2.4 bpm (−7.0; 2.3, *p* = 0.33). The attenuation of HR elevation by ivabradine was significant, *p* < 0.01. Salbutamol alone increased FEV_1_ by 6.0% (2.7; 9.3, *p* < 0.01). This effect was not impaired by ivabradine (FEV_1_ increase by 7.7% (2.8; 12.6, *p* < 0.01 *versus* baseline, *p* = 0.5 *versus* no ivabradine). Ivabradine 5 mg per os prevents heart rate acceleration after inhalation of 400 mg salbutamol. Ivabradine has no impact on lung function in patients with moderate-to-very-severe COPD and CHD comorbidity.

## 1. Introduction

Coronary heart disease (CHD) is found in 18.7–62.8% of patients with chronic obstructive pulmonary disease (COPD) [1]. Patients with concomitant COPD and CHD have an increased risk of hospitalization, cardiovascular complications and mortality [2,3]. It turn, bronchodilator therapy with β_2_-agonist in COPD increases the risk of cardiac arrhythmias [4,5,6]. It is known that single inhalation of salbutamol causes a dose-dependent increase in heart rate (HR) up to 9 beats/min [4]. In patients with COPD and CHD tachycardia increases myocardial oxygen demand and may provoke myocardial ischemia, including silent ischemia [7].

Optimal treatment of combination of COPD and CHD till now is not entirely clear. The use of β-blockers is limited in COPD because of their increase in airway hyperresponsiveness [1,2]. In the real practice clinicians still hesitate to start patients with COPD or asthma on beta-blockers due to the fear of bronchoconstriction [8] in spite of the clear evidences of their efficacy and safety in COPD patients [6]. A promising alternative in this case could be an I_f_ blocker such as ivabradine, which is currently being used with antiischemic target for treatment of CHD [9,10]. The drug selectively acts on the pacemaker cells of the sinus node and decreases HR up to 10 beats/min [11], reducing myocardial oxygen demand and preventing angina pectoris [12].

The aim of the study was to evaluate the protective effect of the I_f_-inhibitor ivabradine on HR acceleration caused by the inhaled short-acting beta2-agonists salbutamol in patients with COPD and CHD.

## 2. Experimental Section

**Figure 1:**
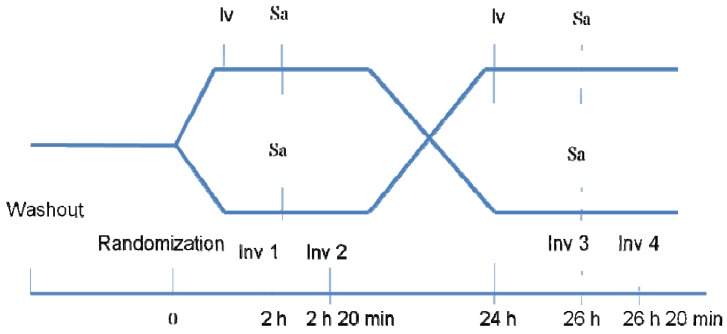
Study design. (Iv: ivabradine; Sa: Salbutamol; Inv: investigation).

In the prospective, short-term, crossover, randomized open-label study (Figure 1) patients with COPD of stage II-IV and concomitant stable CHD with angina pectoris NYHA I-III were included. Patients with HR below 60 beats/min, NYHA IV, current or previous myocardial infarction, atrial fibrillation, frequent extrasystoles, AV-block, diabetes mellitus, malignancy and decompensation of other diseases were not enrolled in the study.

Each patient attended two standard bronchial obstruction reversibility tests separated by at least 24 h to eliminate the possibility of carryover effects from the previous test. One of the tests was performed with combination salbutamol+ivabradine (S+I) and another test was performed with salbutamol (S) alone. The order of administration of test drugs was randomized with the help of a random number table. Accordingly, all patients were divided into two groups with 0.5 probability. Patients of group I were prescribed 5 mg ivabradine (Procoralan, Servier, Neuilly-sur-Seine, France) per os 3 h before salbutamol inhalation on the first day of the study and patients of group II received 5 mg ivabradine only on the second day of the study. A 3 h interval between ivabradine ingestion and salbutamol inhalation was determined to achieve the maximum effect of ivabradine at the time of the test. The patients did not take inhaled bronchodilators and short-acting theophylline 8 h before the test and long-acting agents 12 h before the test.

The study was approved by the local Committee on Biomedical Ethics of the Bashkir State Medical University, Ufa, Russian Federation. All patients provided informed consent.

The patients fasted in the morning of the investigation. Respiratory function was assessed via spirometry. Vital capacity (VC) and forced expiratory volume in the first second (FEV_1_) were estimated in % predicted. HR was measured with pulse oximeter WristOx 3100 (NONIN, Plymouth, MN, USA). Systolic and diastolic blood pressure (SBP and DBP) were measured via the Korotkov method. The systolic pulmonary arterial pressure (SPAP) was measured indirectly via echocardiography. Exercise tolerance was assessed with the 6-minute walk test.

All measurements were made at baseline and 20 min after inhalation of 4 single doses (400 mcg) of salbutamol (Ventolin, GSK, Brentford, Middlesex, UK) via an aerosol inhaler and spacer in a sitting position of the patient between 10-00 and 12-00 a.m.

Data are presented as median (interquartile range) or as mean (95% confidential interval). Differences between the two groups were assessed with non-parametric Mann-Whitney U test, the group dynamics were assessed with Student’s *t*-test for paired samples. A value of *p* < 0.05 was considered to be significant.

## 3. Results

Twenty patients (18 men and two women, aged 43 to 76 years) with COPD in combination with CHD hospitalized at the pulmonology department of Ufa clinical hospital N21 were enrolled in the study. All patients were in stable clinical state. Diagnosis of COPD was established according to the GOLD recommendations.COPD stage II was presented in 8, III—7, and IV—in four patients (Table 1). At the time of the study 14 patients actively smoked, five were former smokers who had stopped smoking more than 6 months ago and one patient had never smoked at all. 35% of the patients had pulmonary hypertension with a SPAP >40 mmHg.

**Figure 2:**
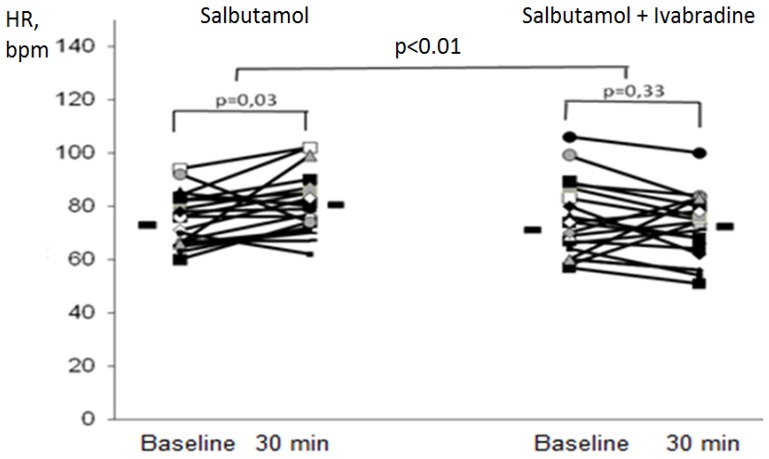
HR dynamics after the inhalation of salbutamol (group S) and salbutamol after ivabradine 5 mg use (group S+I).

In the S group salbutamol inhalation increased HR by 5.5 bpm (0.8; 10.2, *p* = 0.03) (Figure 2), while in the S+I group HR did not change significantly after salbutamol inhalation (−2.4 bpm; CI −7.0; 2.3, *p* = 0.33). The different impact of salbutanol on HR with *versus* without ivabradine pre-treatment was significant (*p* < 0.01). In both, the S and S+I group BP did not change (*p* = 0.5).

The initial values of HR, blood pressure (BP) and lung function indices in groups S and S+I did not differ significantly (Table 2).

In the S group salbutamol inhalation caused a FEV_1_ increase by 6.0 (2.7; 9.3) % pred., *p* < 0.01 (Table 3). Similarly, salbutamol improved FEV1 in the S+I group by 7.7 (2.8; 12.6) % pred., *p* < 0.01, with no significant difference between both treatment groups (*p* = 0.5).

**Table 1:** Characteristics of patients.

Parameters	Values
Age, years	62.0 (57.0–72.0)
BMI, kg/m^2^	26.3 (21.1–28.5)
Smoking, packs × years	40.0 (30.0–73.5)
Postbronchodilatory FEV_1_, % pred.	46.7 (26.0–67.1)
SPAP, mmHg	32.5 (26.0–39.0)
6-min. walktest, m	439 (411–480)
Heart rate, bpm	77 (66–87)
SAP, mmHg	128 (118–146)
DAP, mmHg	80 (72–90)

Data are expressed as median (1st quartile–3rd quartile), BMI = body mass index, FEV_1_ = 1 second forced expiratory volume, SPAP = systolic pulmonary artery pressure, SAP = systolic arterial pressure, DAP = diastolic arterial pressure.

**Table 2:** Baseline characteristics of randomized groups of patients.

	Group I		Group II	
	Salbutamol	Ivabradine+Salbutamol	Ivabradine+Salbutamol	Salbutamol
Heart rate, bpm	78 (69-90)	79 (69-86)	74 (62-84)	76 (67-82)
SAP, mm Hg	124 (120-136)	120 (115-130)	128 (125-144)	130 (120-148)
DAP, mm Hg	80 (74-80)	84 (74-90)	84 (80-90)	86 (70-94)
FEV_1_, % pred.,	39.6 (24.3-67.2)	31,1 (25.7-65.3)	43.4 (29.6-63.6)	48.4 (28.5-52.5)

Data are expressed as median (1st quartile-3rd quartile).

**Table 3:** Changes after salbutamol inhalation *versus* combination ivabradine+salbutamol.

	Salbutamol	Ivabradine+Salbutamol
Heart rate, bpm	5.5 (0.8; 10.2) *	−2.4 (−7.0; 2.3)
SAP, mm Hg	0.3 (−3.5; 4.2)	−2.0 (−7.8; 3.8)
DAP, mm Hg	−1.3 (−4.1; 1.4)	−2.0 (−5.4; 1.4)
FEV_1_, % pred.	6.0 (2.7; 9.3) **	7.7 (2.8; 12.6) **

Data are expressed as mean (95% confidence interval), * *p* < 0.03, ** *p* < 0.01 *versus* baseline.

## 4. Discussion

Combination of COPD and CHD worsens prognosis of the patients and may restrict both bronchodilator and antianginal therapy. A typical side effect of salbutamol use is sinus tachycardia which appears due to direct stimulation of β_2_-adrenergic receptors of the sinus node. In patients with CHD tachycardia increases myocardial oxygen demand and can aggravate myocardial ischemia and even cause myocardial infarction [2,6]. In our study salbutamol at the dose of 400 micrograms increased HR by 5.5 beats/min.

The use of β blockers in patients with COPD and cardiovascular disease has been shown to reduce mortality [5,13] and they are an essential part of CHD basic therapy with well-evidenced antianginal effects in which negative chronotropic action plays a key role. But the use of nonselective beta-blockers in comorbide COPD is limited because of physicians’ fear of bronchoconstriction based on some clinical reports [14,15], idea of “competition” with beta2-agonists [16] and some tertiary literature sources. Some authors also state that beta-blocker use in such case should be probably in lower doses [17] and titrated slowly with attention to lung function and symptoms [14]. That is why clinical studies with negative chronotropic drugs in such comorbidity make sense.

Optimal medication for the patients with such combined pathology should lower HR without any impact on respiratory function. The I_f_ blocker ivabradine best satisfies these conflicting requirements. It is an effective antianginal drug in patients with CHD in combination with COPD that significantly reduces frequency of angina pectoris and duration of silent myocardial ischemia [7]. Also the drug seems to optimize the ventilation-perfusion ratio, increase exercise tolerance, reduce pulmonary artery pressure and has no negative impact on respiratory function [18].

The results of this study showed that under the action of the f-channel blocker ivabradine 5 mg per os salbutamol at the dose of 400 micrograms, as often used in clinical practice, has no effect on HR. The data confirm the results of previous experimental studies [2]. Guth and Dietze [19] found that the block of pacemaker f-channels by another I_f_-blocker zetabradin significantly limits the development of tachycardia during intravenous infusion of the β-agonist isoproterenol and norepinephrine.

The mechanism of functional antagonism between the I_f_-blockers and β-adrenergic activation of the sinus node of the heart is not entirely clear. Electrophysiological studies on the isolated cells have shown that the ionic current through the f-channels is directly modulated by intracellular cyclic adenosinemonophosphate (cAMP) and does not depend on activation of protein kinase A [20]. It is assumed that β-adrenergic modulation of HR is carried by intracellular cAMP and subsequent interaction with f-channels. Thus, blockade of ionic current through the f-channels can effectively prevent the increase in HR after inhalation of β-adrenoreceptor agonists.

Due to the high sinus node selectivity of ivabradine it does not attenuate the bronchodilatator effect of salbutamol. The increase in FEV_1_ after inhalation of salbutamol was not impaired by the prior use of ivabradine. Also BP remained unchanged.

## 5. Conclusions

In patients with moderate-to-very-severe COPD and CHD NYHA I-III ivabradine 5 mg per os effectively prevented the positive chronotropic effect of inhaled salbutamol 400 mcg and did not affect respiratory function and blood pressure.
